# Emerging colistin resistance in *Salmonella enterica* serovar Newport isolates from human infections

**DOI:** 10.1080/22221751.2020.1733439

**Published:** 2020-03-03

**Authors:** Mohammed Elbediwi, Hang Pan, Silpak Biswas, Yan Li, Min Yue

**Affiliations:** a Institute of Preventive Veterinary Sciences and Department of Veterinary Medicine, Zhejiang University College of Animal Sciences, Hangzhou, People’s Republic of China; b Zhejiang Provincial Key Laboratory of Preventive Veterinary Medicine, Hangzhou, People’s Republic of China

**Keywords:** *Salmonella* Newport, antimicrobial resistance, colistin resistance, *mcr-1*, seafood

## Abstract

Worldwide emergence of *Salmonella* enterica serovar Newport (*S*. Newport) infection in humans, in parallel with a significant increasing prevalence of antimicrobial resistance (AR), is a serious public health concern. However, the prevalence of *S*. Newport resistance in China remains largely unknown. A retrospective study of 287 *S.* Newport clinical isolates collected during 1997–2018 was undertaken for characterization of AR profiles using the micro-dilution assay. We found a recent emergence of colistin resistance in four Chinese clinical isolates, including *mcr*-1-positive isolates. Importantly, phylogenomic and microbiological investigations indicate multiple independent clonal transmission of colistin-resistant *S.* Newport isolates of different seafood origins. Our study highlights potential reservoirs for transmission of colistin resistance and suggests that the global food supply chain may facilitate this dissemination.

*Salmonella enterica* serovar Newport (*S.* Newport) is one of the top-ranked serovars for human infections in the USA [1] and the European countries [2], while its prevalence in China remains obscure. Antimicrobial resistance (AR) has been thought to play significant roles in the dissemination of bacterial pathogen [3]. Recently, several countries have reported an increasing prevalence of resistance in *S*. Newport isolates from animals and humans [3–5]. However, limited information is available on the resistance of Chinese *Salmonella* Newport isolates to antimicrobials, including the last-resort drug colistin. Here, we report a retrospective study in AR prevalence of *S.* Newport isolates recovered from Chinese patients, with a focus on genetic determinants for colistin resistance.

A total of 287 human clinical *Salmonella* Newport isolates from faecal samples of patients with diarrheal diseases (all sporadic cases) and clinically healthy carriers, were obtained by the Centers for Disease Control and Prevention (CDC) of nine municipal cities or provinces (Shanghai, Zhejiang, Chongqing, Fujian, Hubei, Shanxi, Yunnan, Guanxi and Shenzhen) in China between 1997 and 2018 (Supplemental Table 1). All strains were subjected to susceptibility testing by standard broth micro-dilution assay using 14 antimicrobial agents (Supplemental Figure 1), and the results were interpreted according to Clinical & Laboratory Standards Institute (CLSI, 2019). Seventy-eight per cent (*n* = 224) of the isolates were resistant to at least one antimicrobial drug. Over half of the isolates (55.7%) were resistant to one or two antimicrobial drugs, and the frequency of multidrug-resistant (MDR) strains with resistance to at least three different antimicrobial drugs of different classes was 21.5% (Supplemental Figure 1), slightly higher than the previous studies on *S.* Newport in Shanghai (15.2%) [5] and the USA (8%) [3].

Notably, between 2015 and 2017, four isolates (1.4%) from Guangxi and Zhejiang provinces were found to have higher minimum inhibition concentration values of colistin (4–16 mg/L). Of the four strains, three were isolated from patients with diarrhoea, one from asymptomatic carrier via health examination. We further used multiplex PCR [6] to detect the presence of *mcr* genes in colistin-resistant strains and found *Sal_276* and *Sal_311* carry *mcr*-*1* genes (Supplemental Figure 2). Successful conjugal transfer from *mcr-1-*positive strains to the recipient *Escherichia coli* j53 (streptomycin- and rifampicin-resistant) was accomplished at 25 and 37°C in liquid medium as well as on solid agar plates [7]. Whole-genome sequencing was performed on the Illumina MiSeq platform, and paired-end reads were assessed, filtered with fastq-mcf tool (http://code.google.com/p/ea-utils), and assembled with SPAdes v. 3.12 (http://cab.spbu.ru/software/spades/). Multilocus sequence typing (MLST), detection of resistance genes and the chromosomal mutations in *mgrB, phoP/phoQ, pmrA, pmrB*, that confer resistance to colistin, were analysed by using the Center for Genomic Epidemiology platform (https://cge.cbs.dtu.dk/services/). For the two strains without the *mcr-1* gene, we found that two chromosomal mutations in *pmrA* (Valine to Phenylalanine at position 30 and Alanine to Valine at position 33) were detected in *Sal_354*, and one mutation in *pmrB* (Alanine to Serine at position 181) was detected in *Sal_353*. These two isolates also carry an additional *eptC* gene, which is known *mcr-1* homologue [8] (Supplemental Table 3). The presence of these novel chromosomal mutations in *pmrA* and *pmrB*, as well as *eptC*, are likely responsible for the colistin resistance in *mcr*-negative isolates.

In terms of additional resistance genes in colistin-resistant isolates, all strains harboured *aac(6’)-Iaa* gene which encodes resistance to aminoglycosides, particularly for gentamicin [9]. Resistance to aminoglycosides was also mediated by *aph(*3’*)-Ia*, *aph(4)-Ia* and *aac(3)-Iva* in strains *Sal*_276 and *Sal*_311. The *aac(6’)Ib-cr* gene responsible for resistance to aminoglycosides, in addition to quinolones, was found only in *Sal*_354. Importantly, plasmid-mediated quinolone resistance genes (*qnrS1* and *qnrA1*) were detected in *Sal*_276 and *Sal*_354, respectively. Genes encoding β-lactamases were identified in two strains (*Sal*_311 carried *bla*_TEM-1C_ and *Sal*_354 carried *bla*_OX-1_). The gene *Sul2* which encodes resistance to sulfonamide was only found in *Sal*_354 (Supplemental Table 3).

For further analysis of the *mcr-1-*carrying plasmids, the plasmid sequences were recovered from whole genomic sequences by using Plasmid SPAdes [10] and PLACNETw [11]. Both analytic pipelines delivered the identical results. Two strains carried IncHI2-like plasmids with the IncF transfer region (Figure 1(a), Supplemental Figure 2). These two plasmids showed highest sequence identity to a typical IncHI2-type backbone plasmid (Figure 1(a), Supplemental Figure 2). The IncHI2/IncF plasmid type was also detected in *S*. Typhimurium isolated from pig faecal sample [12] and from the food sample [13]. Interestingly, a PAP2 family protein that is frequently associated with *mcr-1* was located directly downstream of *mcr-1* (Figure 1(b)). The insertion sequence (IS) 5 and hypothetical protein were located upstream of the *mcr-1* gene in *SAL_276*. However, IS1 was found in the downstream of *SAL_311* (Figure 1(b)). To the best of our knowledge, this is the first detection of *mcr-1* gene in *S*. Newport in humans in China, even though *mcr*-*1* has been previously reported in *S*. Newport isolates from pigs and chicken in Italy [14,15], and China [16,17].

**Figure 1.**
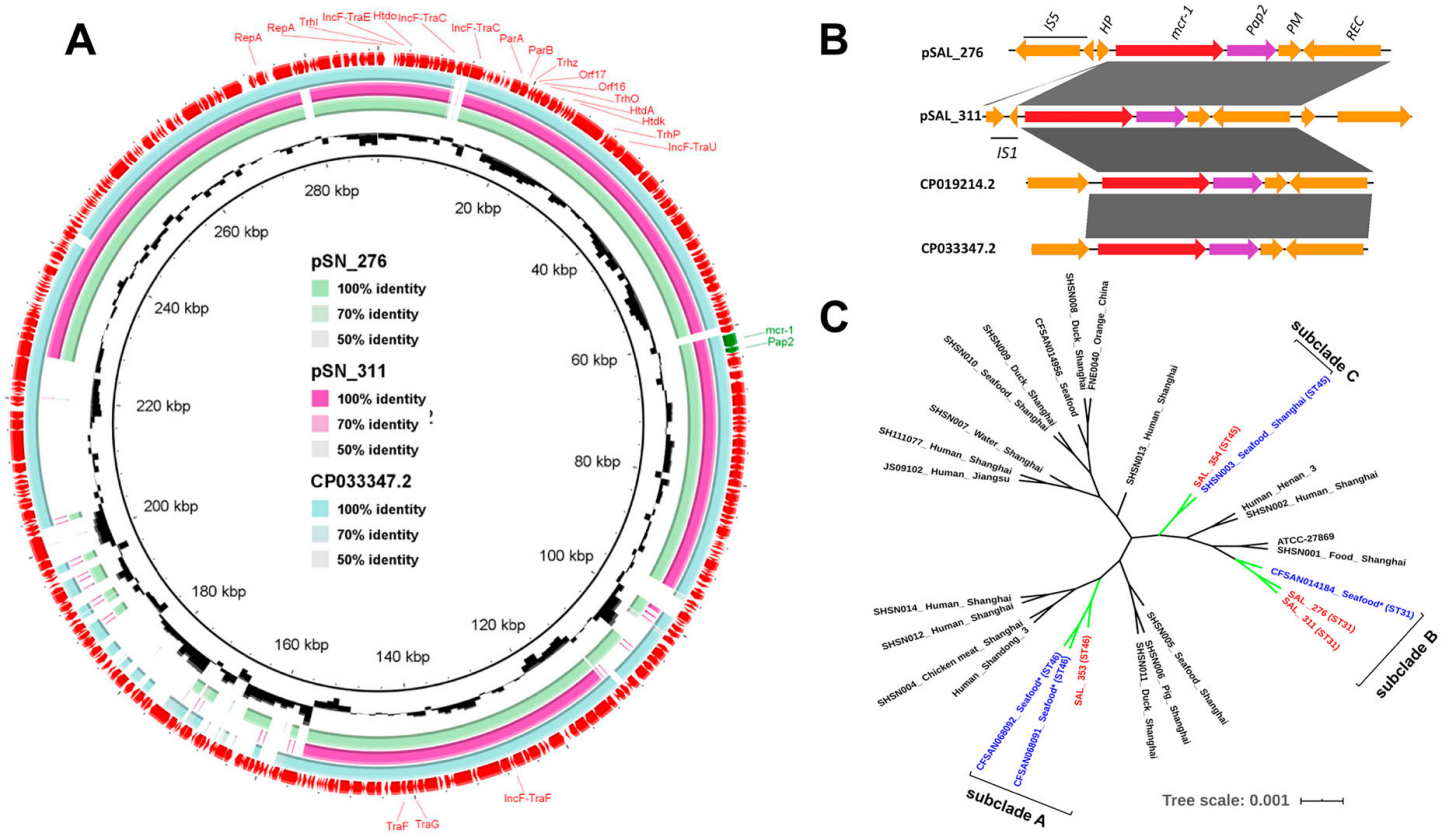
Comparative sequence analysis of the *mcr*-carrying plasmids and phylogenomic features for all Chinese *Salmonella* Newport genomes available in the NCBI database in addition to four new colistin-resistant strains in this study. (a) Sequence comparison of two reconstructed *mcr*-1-positive plasmids from whole-genome sequence. The out layer circle refer to the CP01924.2 plasmid. (b) Genetic backbone flanking with *mcr*-1 sequence of *SAL*_*276* and *SAL*_*311* (CP019214.2 in *E. coli* isolated from sewage in China and CP033347.2 in *S*. Typhimurium isolated from pork in China). IS, insertion sequence; HP, hypothetical protein; PM, putative membrane; REC, recombinase. (c) Phylogenomic analysis of all available *S*. Newport Chinese isolates in this study, including 24 additional Chinese *S*. Newport isolates from different hosts retrieved from the NCBI database. The four isolates examined in this study in addition to their closely related seafood-origin isolates were clustered together in the three subclades (A, B and C). Tree scale represents the genetic distance between the isolates used to construct the tree. *These isolates were collected by US FDA, mentioning China as their place of origin.

To further investigate the possible source of colistin-resistant *S*. Newport, we collected all available *S*. Newport genomes of Chinese origin from the NCBI database (*n* = 24) and one from the USA (ATCC 27869) as a reference, in the phylogenomic analysis (Supplemental Table 2). Whole genomic SNPs calling was performed using Snippy v4.4.4 (https://github.com/tseemann/snippy). Phylogenetic trees were constructed by the maximum likelihood method with RAxML model. We confirmed the polygenetic feature in *S*. Newport, and found that colistin-resistant strains were independently clustered together with Chinese seafood isolates in three different subclades, with the same STs in each subclade (Figure 1(c)). In subclade A, we found two Chinese isolates (CFSAN068092 and CFSAN068092), both recovered from frozen small octopus exported to the USA, were grouped together with a chromosome-mediated colistin-resistant strain *Sal_353* in the same year of 2017. Subclade B suggested that *Salmonella* Newport isolate (CFSAN014184), recovered in 2004 from an exported tilapia sample, was grouped together with two *mcr*-carrying strains in this study. Importantly, the Chinese costal region Guangxi, where two *mcr*-positive strains were recovered, is the world largest producer of tilapia, with 20% exported to the USA, raising significant concerns in the context of the global food supply chain. Subclade C indicated a local linkage with the turtle origin in 2011, which is occasionally served as food for the Chinese. Further analysis showed that these Chinese seafood isolates were closely related to human isolates and contained one chromosomal mutations in *phoPQ* (Methionine to Valine at position 1), in addition to *eptA (pmrD)* and *eptC* genes (*mcr-1* homologues), that confer resistance to colistin in all four isolates. Both *mcr*-positive (ST31) and chromosome-mediated (ST46) strains have clonal linkage with seafood origin, including tilapia and octopus. The host or sampling origin was significantly associated with the particular ST, which was suggested by our previous large-scale population study [18]. We also found an isolate from ST45, a previous bovine adaptive subclade, with colistin resistance. Interestingly, this particular strain (*Sal_354*) might have link with a turtle isolate in the local food market. Considering Guangxi and Zhejiang provinces were top seafood producers in China due to favourable coastal locations [19], our study provided the evidence that multi-independent seafood, a previous underestimated player in foodborne transmission, could serve as the reservoir for human *Salmonella* Newport infections and critical AR dissemination.

Since colistin is not officially allowed in Chinese aquaculture, the aquatic environment is liable to contamination through colistin-treated animal faeces via the use of manures in feeding farmed fish. It is likely that the polygenetic feature of *S.* Newport may expand its capability in *mcr* dissemination [20,21], and more importantly, global food chain may further facilitate the dissemination of foodborne pathogens and AR dissemination. Appropriate regulations for monitoring AR of the imported food are urgently needed, particularly, for regions with AR prevalence.

Although this study could not determine the prevalence of *S*. Newport isolates due to lack of the information on the total number of samples for bacterial isolation, the total number of *Salmonella* isolates, our findings demonstrated a multi-independent dissemination of colistin-resistant *S*. Newport for human infections in China. We also found these human isolates harbouring *mcr-1* were closely related to colistin-resistant exported seafood isolates from coastal provinces of China that are important seafood exporters. However, the direct evidence for seafood-mediated transmission is required in further investigation. We highlight that the use of antimicrobial in aquaculture should be better assessed and regulated, in parallel with land livestock in China [22,23], and an enhanced integrated Chinese surveillance system is warranted for including aquaculture products, community, and hospitals.

## Supplementary Material

Supplemental Material

## Data Availability

Raw sequencing reads of *mcr-1-* carrying strains have been deposited in the NCBI BioProject database under accession numbers PRJNA573539.
