# Supplementary material for: Emerging colistin resistance in Salmonella enterica serovar Newport isolates from human infections
Source: Emerg Microbes Infect. 2020 Mar 3;9(1):535–8. doi: 10.1080/22221751.2020.1733439 (PMC7067173; doi:10.1080/22221751.2020.1733439)
Supplement: Supplemental Material [file TEMI_A_1733439_SM4621.docx]

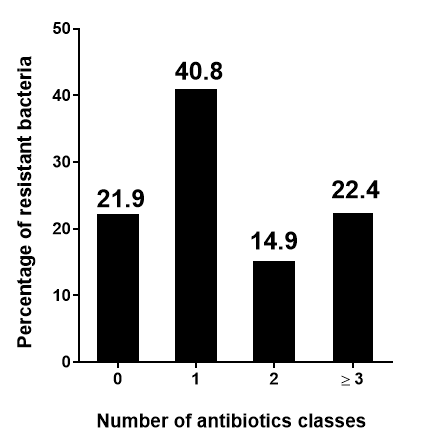


**Supplemental Figure 1**. **Frequency and distribution of resistance to antimicrobial agents among 287 *S*. Newport isolates obtained from clinical specimens in China between 1997 and 2018.** Occurrence of multidrug resistance among 287 *S.* Newport. The *x*-axis indicates the number of antimicrobial classes. The number on top of each column represents the percentage of resistant isolates to different numbers of antimicrobial agents. (Strains were also categorized into three antibiotic categories: pan‐susceptible, simple‐drug‐resistance (SDR) defined as one or two types of antimicrobial classes, or multi‐drug‐resistance (MDR) defined as resistance to drugs of at least three different antimicrobial classes. The antimicrobials used includes ampicillin; gentamicin; kanamycin, tetracycline; ciprofloxacin; chloramphenicol; sulfamethoxazole-trimethoprim; amoxicillin-clavulanic acid; ceftriaxone; cefoxitin; nalidixic acid; colistin; meropenem.


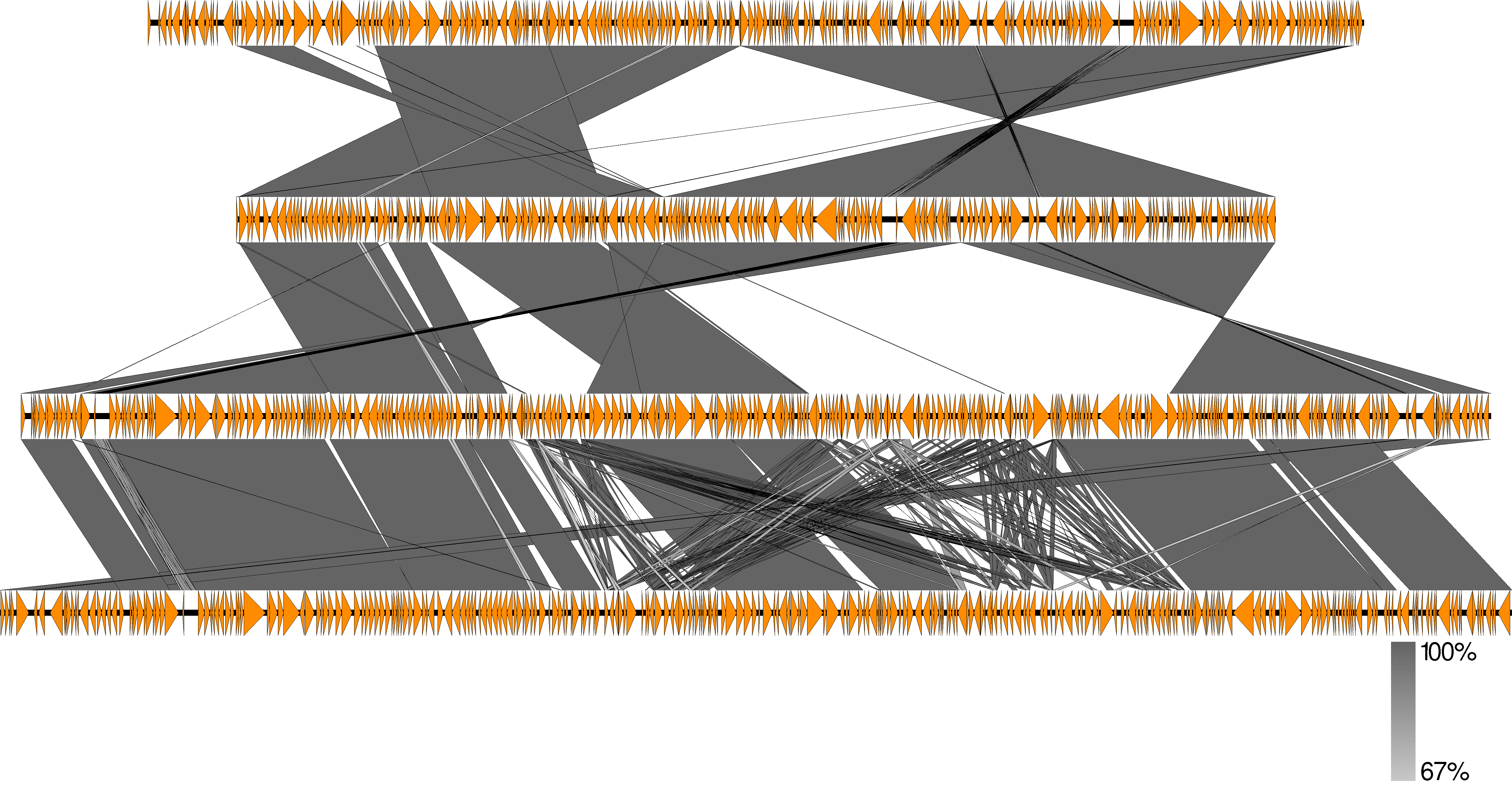


pSN_276

pSN_311

CP019214.2

CP033347.2

*mcr-1*

Toxin Higb

IncF-TraU

IncF-TraC

IncH2- TrhO

IncF-TraF

IncF-TraC

IncH2-Htdo

IncF-TraE

*Pap2*

IncH2-Trhl

*RepA*

**Supplemental Figure 2.** **Comparative plasmid sequence among the two plasmids pSN_276 and pSN_311 and the reference sequences of plasmids CP019214.2, and CP033347.2**. Areas shaded in gray indicate homologies between the corresponding genetic loci on each plasmid. The Open Reading Frames are shown in arrow.

**Supplemental Table 1. Metadata for the examined Chinese *S.* Newport isolates**

| **Isolate (ID)** | **Isolation place** | **Host** | **Resistome** | **Year** | **Laboratory Code** |
| --- | --- | --- | --- | --- | --- |
| **SAL_1** | **Shanghai** | **Human** | **Pan-susceptible** | **2000** | **SAL00001** |
| **SAL_2** | **Shanghai** | **Human** | **Pan-susceptible** | **2000** | **SAL00002** |
| **SAL_3** | **Shanghai** | **Human** | **Pan-susceptible** | **2000** | **SAL00003** |
| **SAL_14** | **Shanghai** | **Human** | **MDR** | **2000** | **SAL00014** |
| **SAL_15** | **Shanghai** | **Human** | **Pan-susceptible** | **2002** | **SAL00015** |
| **SAL_16** | **Shanghai** | **Human** | **SDR** | **2002** | **SAL00016** |
| **SAL_17** | **Shanghai** | **Human** | **SDR** | **2002** | **SAL00017** |
| **SAL_18** | **Shanghai** | **Human** | **Pan-susceptible** | **2002** | **SAL00018** |
| **SAL_19** | **Shanghai** | **Human** | **Pan-susceptible** | **2002** | **SAL00019** |
| **SAL_27** | **Zhejiang** | **Human** | **SDR** | **2017** | **SAL00027** |
| **SAL_28** | **Zhejiang** | **Human** | **SDR** | **2017** | **SAL00028** |
| **SAL_29** | **Zhejiang** | **Human** | **SDR** | **2017** | **SAL00029** |
| **SAL_30** | **Zhejiang** | **Human** | **Pan-susceptible** | **2017** | **SAL00030** |
| **SAL_31** | **Zhejiang** | **Human** | **SDR** | **2017** | **SAL00031** |
| **SAL_32** | **Zhejiang** | **Human** | **MDR** | **2017** | **SAL00032** |
| **SAL_33** | **Zhejiang** | **Human** | **MDR** | **2017** | **SAL00033** |
| **SAL_34** | **Zhejiang** | **Human** | **MDR** | **2017** | **SAL00034** |
| **SAL_35** | **Zhejiang** | **Human** | **MDR** | **2017** | **SAL00035** |
| **SAL_36** | **Zhejiang** | **Human** | **MDR** | **2016** | **SAL00036** |
| **SAL_37** | **Shanghai** | **Human** | **MDR** | **2017** | **SAL00037** |
| **SAL_38** | **Shanghai** | **Human** | **SDR** | **2017** | **SAL00038** |
| **SAL_39** | **Shanghai** | **Human** | **SDR** | **2017** | **SAL00039** |
| **SAL_40** | **Shanghai** | **Human** | **MDR** | **2017** | **SAL00040** |
| **SAL_41** | **Shanghai** | **Human** | **SDR** | **2017** | **SAL00041** |
| **SAL_42** | **Shanghai** | **Human** | **SDR** | **2017** | **SAL00042** |
| **SAL_43** | **Shanghai** | **Human** | **MDR** | **2017** | **SAL00043** |
| **SAL_44** | **Shanghai** | **Human** | **Pan-susceptible** | **2017** | **SAL00044** |
| **SAL_45** | **Shanghai** | **Human** | **Pan-susceptible** | **2017** | **SAL00045** |
| **SAL_46** | **Shanghai** | **Human** | **MDR** | **2017** | **SAL00046** |
| **SAL_47** | **Shanghai** | **Human** | **Pan-susceptible** | **2017** | **SAL00047** |
| **SAL_48** | **Shanghai** | **Human** | **SDR** | **2017** | **SAL00048** |
| **SAL_49** | **Shanghai** | **Human** | **MDR** | **2017** | **SAL00049** |
| **SAL_50** | **Shanghai** | **Human** | **Pan-susceptible** | **2017** | **SAL00050** |
| **SAL_51** | **Fujian** | **Human** | **SDR** | **2017** | **SAL00051** |
| **SAL_52** | **Fujian** | **Human** | **MDR** | **2017** | **SAL00052** |
| **SAL_53** | **Fujian** | **Human** | **Pan-susceptible** | **2017** | **SAL00053** |
| **SAL_54** | **Fujian** | **Human** | **Pan-susceptible** | **2017** | **SAL00054** |
| **SAL_55** | **Fujian** | **Human** | **SDR** | **2017** | **SAL00055** |
| **SAL_56** | **Fujian** | **Human** | **SDR** | **2017** | **SAL00056** |
| **SAL_57** | **Fujian** | **Human** | **Pan-susceptible** | **2017** | **SAL00057** |
| **SAL_58** | **Fujian** | **Human** | **MDR** | **2017** | **SAL00058** |
| **SAL_59** | **Fujian** | **Human** | **Pan-susceptible** | **2017** | **SAL00059** |
| **SAL_60** | **Fujian** | **Human** | **MDR** | **2017** | **SAL00060** |
| **SAL_61** | **Fujian** | **Human** | **Pan-susceptible** | **2017** | **SAL00061** |
| **SAL_62** | **Fujian** | **Human** | **MDR** | **2017** | **SAL00062** |
| **SAL_63** | **Fujian** | **Human** | **SDR** | **2017** | **SAL00063** |
| **SAL_64** | **Fujian** | **Human** | **Pan-susceptible** | **2017** | **SAL00064** |
| **SAL_81** | **Shanghai** | **Human** | **MDR** | **2006** | **SAL00081** |
| **SAL_82** | **Shanghai** | **Human** | **SDR** | **2006** | **SAL00082** |
| **SAL_83** | **Shanghai** | **Human** | **Pan-susceptible** | **2006** | **SAL00083** |
| **SAL_84** | **Shanghai** | **Human** | **SDR** | **2006** | **SAL00084** |
| **SAL_85** | **Shanghai** | **Human** | **Pan-susceptible** | **2006** | **SAL00085** |
| **SAL_86** | **Shanghai** | **Human** | **SDR** | **2006** | **SAL00086** |
| **SAL_87** | **Shanghai** | **Human** | **SDR** | **2006** | **SAL00087** |
| **SAL_88** | **Shanghai** | **Human** | **Pan-susceptible** | **2006** | **SAL00088** |
| **SAL_89** | **Shanghai** | **Human** | **Pan-susceptible** | **2006** | **SAL00089** |
| **SAL_90** | **Shanghai** | **Human** | **SDR** | **2007** | **SAL00090** |
| **SAL_91** | **Shanghai** | **Human** | **SDR** | **2007** | **SAL00091** |
| **SAL_92** | **Shanghai** | **Human** | **SDR** | **2007** | **SAL00092** |
| **SAL_93** | **Shanghai** | **Human** | **SDR** | **2007** | **SAL00093** |
| **SAL_94** | **Shanghai** | **Human** | **SDR** | **2007** | **SAL00094** |
| **SAL_95** | **Shanghai** | **Human** | **SDR** | **2007** | **SAL00095** |
| **SAL_96** | **Shanghai** | **Human** | **SDR** | **2007** | **SAL00096** |
| **SAL_97** | **Shanghai** | **Human** | **SDR** | **2008** | **SAL00097** |
| **SAL_98** | **Shanghai** | **Human** | **Pan-susceptible** | **2008** | **SAL00098** |
| **SAL_99** | **Shanghai** | **Human** | **MDR** | **2008** | **SAL00099** |
| **SAL_100** | **Shanghai** | **Human** | **MDR** | **2008** | **SAL00100** |
| **SAL_101** | **Shanghai** | **Human** | **MDR** | **2008** | **SAL00101** |
| **SAL_105** | **Shanghai** | **Human** | **MDR** | **2009** | **SAL00105** |
| **SAL_106** | **Shanghai** | **Human** | **MDR** | **2009** | **SAL00106** |
| **SAL_107** | **Shanghai** | **Human** | **MDR** | **2009** | **SAL00107** |
| **SAL_108** | **Shanghai** | **Human** | **MDR** | **2009** | **SAL00108** |
| **SAL_109** | **Shanghai** | **Human** | **Pan-susceptible** | **2009** | **SAL00109** |
| **SAL_110** | **Shanghai** | **Human** | **MDR** | **2009** | **SAL00110** |
| **SAL_111** | **Shanghai** | **Human** | **MDR** | **2009** | **SAL00111** |
| **SAL_112** | **Shanghai** | **Human** | **MDR** | **2009** | **SAL00112** |
| **SAL_113** | **Shanghai** | **Human** | **SDR** | **2009** | **SAL00113** |
| **SAL_118** | **Shanghai** | **Human** | **MDR** | **2010** | **SAL00118** |
| **SAL_119** | **Shanghai** | **Human** | **MDR** | **2010** | **SAL00119** |
| **SAL_120** | **Shanghai** | **Human** | **MDR** | **2010** | **SAL00120** |
| **SAL_121** | **Shanghai** | **Human** | **SDR** | **2010** | **SAL00121** |
| **SAL_122** | **Shanghai** | **Human** | **SDR** | **2010** | **SAL00122** |
| **SAL_123** | **Shanghai** | **Human** | **SDR** | **2010** | **SAL00123** |
| **SAL_124** | **Shanghai** | **Human** | **Pan-susceptible** | **2010** | **SAL00124** |
| **SAL_125** | **Shanghai** | **Human** | **Pan-susceptible** | **2010** | **SAL00125** |
| **SAL_126** | **Shanghai** | **Human** | **Pan-susceptible** | **2010** | **SAL00126** |
| **SAL_127** | **Shanghai** | **Human** | **Pan-susceptible** | **2010** | **SAL00127** |
| **SAL_128** | **Shanghai** | **Human** | **SDR** | **2010** | **SAL00128** |
| **SAL_129** | **Shanghai** | **Human** | **Pan-susceptible** | **2010** | **SAL00129** |
| **SAL_130** | **Shanghai** | **Human** | **SDR** | **2010** | **SAL00130** |
| **SAL_131** | **Shanghai** | **Human** | **SDR** | **2010** | **SAL00131** |
| **SAL_132** | **Shanghai** | **Human** | **SDR** | **2010** | **SAL00132** |
| **SAL_136** | **Shanghai** | **Human** | **Pan-susceptible** | **2011** | **SAL00136** |
| **SAL_137** | **Shanghai** | **Human** | **SDR** | **2011** | **SAL00137** |
| **SAL_138** | **Shanghai** | **Human** | **SDR** | **2011** | **SAL00138** |
| **SAL_139** | **Shanghai** | **Human** | **SDR** | **2011** | **SAL00139** |
| **SAL_140** | **Shanghai** | **Human** | **Pan-susceptible** | **2011** | **SAL00140** |
| **SAL_141** | **Shanghai** | **Human** | **Pan-susceptible** | **2011** | **SAL00141** |
| **SAL_142** | **Shanghai** | **Human** | **SDR** | **2011** | **SAL00142** |
| **SAL_143** | **Shanghai** | **Human** | **SDR** | **2011** | **SAL00143** |
| **SAL_144** | **Shanghai** | **Human** | **SDR** | **2011** | **SAL00144** |
| **SAL_145** | **Shanghai** | **Human** | **Pan-susceptible** | **2011** | **SAL00145** |
| **SAL_146** | **Shanghai** | **Human** | **SDR** | **2011** | **SAL00146** |
| **SAL_147** | **Shanghai** | **Human** | **SDR** | **2011** | **SAL00147** |
| **SAL_148** | **Shanghai** | **Human** | **SDR** | **2011** | **SAL00148** |
| **SAL_149** | **Shanghai** | **Human** | **MDR** | **2011** | **SAL00149** |
| **SAL_153** | **Shanghai** | **Human** | **Pan-susceptible** | **2012** | **SAL00153** |
| **SAL_154** | **Shanghai** | **Human** | **SDR** | **2012** | **SAL00154** |
| **SAL_155** | **Shanghai** | **Human** | **SDR** | **2012** | **SAL00155** |
| **SAL_156** | **Shanghai** | **Human** | **SDR** | **2012** | **SAL00156** |
| **SAL_157** | **Shanghai** | **Human** | **MDR** | **2012** | **SAL00157** |
| **SAL_158** | **Shanghai** | **Human** | **Pan-susceptible** | **2012** | **SAL00158** |
| **SAL_159** | **Shanghai** | **Human** | **MDR** | **2012** | **SAL00159** |
| **SAL_160** | **Shanghai** | **Human** | **MDR** | **2012** | **SAL00160** |
| **SAL_161** | **Shanghai** | **Human** | **SDR** | **2012** | **SAL00161** |
| **SAL_162** | **Shanghai** | **Human** | **MDR** | **2012** | **SAL00162** |
| **SAL_163** | **Shanghai** | **Human** | **SDR** | **2012** | **SAL00163** |
| **SAL_164** | **Shanghai** | **Human** | **SDR** | **2012** | **SAL00164** |
| **SAL_165** | **Shanghai** | **Human** | **SDR** | **2012** | **SAL00165** |
| **SAL_166** | **Shanghai** | **Human** | **Pan-susceptible** | **2012** | **SAL00166** |
| **SAL_167** | **Shanghai** | **Human** | **Pan-susceptible** | **2012** | **SAL00167** |
| **SAL_168** | **Shanghai** | **Human** | **Pan-susceptible** | **2012** | **SAL00168** |
| **SAL_169** | **Shanghai** | **Human** | **SDR** | **2012** | **SAL00169** |
| **SAL_170** | **Shanghai** | **Human** | **Pan-susceptible** | **2012** | **SAL00170** |
| **SAL_171** | **Shanghai** | **Human** | **Pan-susceptible** | **2012** | **SAL00171** |
| **SAL_172** | **Shanghai** | **Human** | **Pan-susceptible** | **2012** | **SAL00172** |
| **SAL_175** | **Shanghai** | **Human** | **Pan-susceptible** | **2013** | **SAL00175** |
| **SAL_176** | **Shanghai** | **Human** | **MDR** | **2013** | **SAL00176** |
| **SAL_177** | **Shanghai** | **Human** | **MDR** | **2013** | **SAL00177** |
| **SAL_178** | **Shanghai** | **Human** | **SDR** | **2013** | **SAL00178** |
| **SAL_179** | **Shanghai** | **Human** | **MDR** | **2013** | **SAL00179** |
| **SAL_180** | **Shanghai** | **Human** | **Pan-susceptible** | **2013** | **SAL00180** |
| **SAL_181** | **Shanghai** | **Human** | **SDR** | **2013** | **SAL00181** |
| **SAL_182** | **Shanghai** | **Human** | **SDR** | **2013** | **SAL00182** |
| **SAL_183** | **Shanghai** | **Human** | **Pan-susceptible** | **2013** | **SAL00183** |
| **SAL_184** | **Shanghai** | **Human** | **Pan-susceptible** | **2013** | **SAL00184** |
| **SAL_185** | **Shanghai** | **Human** | **Pan-susceptible** | **2013** | **SAL00185** |
| **SAL_186** | **Shanghai** | **Human** | **SDR** | **2013** | **SAL00186** |
| **SAL_187** | **Shanghai** | **Human** | **SDR** | **2013** | **SAL00187** |
| **SAL_188** | **Shanghai** | **Human** | **SDR** | **2013** | **SAL00188** |
| **SAL_189** | **Shanghai** | **Human** | **Pan-susceptible** | **2013** | **SAL00189** |
| **SAL_190** | **Shanghai** | **Human** | **MDR** | **2013** | **SAL00190** |
| **SAL_191** | **Shanghai** | **Human** | **Pan-susceptible** | **2013** | **SAL00191** |
| **SAL_192** | **Shanghai** | **Human** | **SDR** | **2013** | **SAL00192** |
| **SAL_193** | **Shanghai** | **Human** | **SDR** | **2013** | **SAL00193** |
| **SAL_194** | **Shanghai** | **Human** | **MDR** | **2013** | **SAL00194** |
| **SAL_195** | **Shanghai** | **Human** | **SDR** | **2013** | **SAL00195** |
| **SAL_196** | **Shanghai** | **Human** | **SDR** | **2013** | **SAL00196** |
| **SAL_197** | **Shanghai** | **Human** | **Pan-susceptible** | **2013** | **SAL00197** |
| **SAL_198** | **Shanghai** | **Human** | **SDR** | **2013** | **SAL00198** |
| **SAL_199** | **Shanghai** | **Human** | **SDR** | **2013** | **SAL00199** |
| **SAL_200** | **Shanghai** | **Human** | **Pan-susceptible** | **2013** | **SAL00200** |
| **SAL_201** | **Shanghai** | **Human** | **SDR** | **2013** | **SAL00201** |
| **SAL_202** | **Shanghai** | **Human** | **SDR** | **2013** | **SAL00202** |
| **SAL_203** | **Shanghai** | **Human** | **MDR** | **2013** | **SAL00203** |
| **SAL_204** | **Shanghai** | **Human** | **Pan-susceptible** | **2013** | **SAL00204** |
| **SAL_205** | **Shanghai** | **Human** | **SDR** | **2013** | **SAL00205** |
| **SAL_206** | **Hubei** | **Human** | **Pan-susceptible** | **2000** | **SAL00206** |
| **SAL_207** | **Hubei** | **Human** | **Pan-susceptible** | **2000** | **SAL00207** |
| **SAL_208** | **Hubei** | **Human** | **MDR** | **2000** | **SAL00208** |
| **SAL_209** | **Hubei** | **Human** | **Pan-susceptible** | **2000** | **SAL00209** |
| **SAL_210** | **Hubei** | **Human** | **Pan-susceptible** | **2000** | **SAL00210** |
| **SAL_211** | **Shanghai** | **Human** | **SDR** | **2013** | **SAL00211** |
| **SAL_212** | **Shanghai** | **Human** | **SDR** | **2013** | **SAL00212** |
| **SAL_213** | **Shanghai** | **Human** | **SDR** | **2013** | **SAL00213** |
| **SAL_214** | **Shanghai** | **Human** | **SDR** | **2013** | **SAL00214** |
| **SAL_230** | **Shanghai** | **Human** | **SDR** | **2014** | **SAL00230** |
| **SAL_231** | **Shanghai** | **Human** | **SDR** | **2014** | **SAL00231** |
| **SAL_232** | **Shanghai** | **Human** | **MDR** | **2014** | **SAL00232** |
| **SAL_233** | **Shanghai** | **Human** | **Pan-susceptible** | **2014** | **SAL00233** |
| **SAL_234** | **Shanghai** | **Human** | **SDR** | **2014** | **SAL00234** |
| **SAL_235** | **Shanghai** | **Human** | **MDR** | **2014** | **SAL00235** |
| **SAL_236** | **Shanghai** | **Human** | **MDR** | **2014** | **SAL00236** |
| **SAL_237** | **Shanghai** | **Human** | **MDR** | **2014** | **SAL00237** |
| **SAL_238** | **Shanghai** | **Human** | **Pan-susceptible** | **2014** | **SAL00238** |
| **SAL_239** | **Shanghai** | **Human** | **Pan-susceptible** | **2007** | **SAL00239** |
| **SAL_240** | **Shanghai** | **Human** | **Pan-susceptible** | **2007** | **SAL00240** |
| **SAL_241** | **Shanghai** | **Human** | **SDR** | **2015** | **SAL00241** |
| **SAL_245** | **Shanghai** | **Human** | **Pan-susceptible** | **2015** | **SAL00245** |
| **SAL_246** | **Shanghai** | **Human** | **MDR** | **2015** | **SAL00246** |
| **SAL_247** | **Shanghai** | **Human** | **SDR** | **2015** | **SAL00247** |
| **SAL_248** | **Shanghai** | **Human** | **Pan-susceptible** | **2015** | **SAL00248** |
| **SAL_249** | **Shanghai** | **Human** | **SDR** | **2015** | **SAL00249** |
| **SAL_250** | **Shanghai** | **Human** | **SDR** | **2015** | **SAL00250** |
| **SAL_251** | **Shanghai** | **Human** | **MDR** | **2015** | **SAL00251** |
| **SAL_252** | **Shanghai** | **Human** | **MDR** | **2015** | **SAL00252** |
| **SAL_253** | **Shanghai** | **Human** | **Pan-susceptible** | **2015** | **SAL00253** |
| **SAL_254** | **Shanghai** | **Human** | **SDR** | **2015** | **SAL00254** |
| **SAL_255** | **Shanghai** | **Human** | **SDR** | **2015** | **SAL00255** |
| **SAL_256** | **Shanghai** | **Human** | **Pan-susceptible** | **2015** | **SAL00256** |
| **SAL_257** | **Shanghai** | **Human** | **Pan-susceptible** | **2015** | **SAL00257** |
| **SAL_258** | **Shanghai** | **Human** | **SDR** | **2015** | **SAL00258** |
| **SAL_259** | **Zhejiang** | **Human** | **SDR** | **2015** | **SAL00259** |
| **SAL_260** | **Fujian** | **Human** | **Pan-susceptible** | **2014** | **SAL00260** |
| **SAL_261** | **Fujian** | **Human** | **Pan-susceptible** | **2015** | **SAL00261** |
| **SAL_262** | **Shanghai** | **Human** | **MDR** | **2015** | **SAL00262** |
| **SAL_263** | **Shanghai** | **Human** | **MDR** | **2015** | **SAL00263** |
| **SAL_264** | **Shanghai** | **Human** | **MDR** | **2015** | **SAL00264** |
| **SAL_265** | **Chongqing** | **Human** | **MDR** | **2014** | **SAL00265** |
| **SAL_270** | **Guangxi** | **Human** | **MDR** | **2016** | **SAL00270** |
| **SAL_271** | **Guangxi** | **Human** | **MDR** | **2016** | **SAL00271** |
| **SAL_272** | **Guangxi** | **Human** | **MDR** | **2016** | **SAL00272** |
| **SAL_273** | **Guangxi** | **Human** | **MDR** | **2016** | **SAL00273** |
| **SAL_274** | **Guangxi** | **Human** | **MDR** | **2016** | **SAL00274** |
| **SAL_275** | **Guangxi** | **Human** | **MDR** | **2015** | **SAL00275** |
| **SAL_276** | **Guangxi** | **Human** | **MDR** | **2015** | **SAL00279** |
| **SAL_277** | **Guangxi** | **Human** | **MDR** | **2015** | **SAL00277** |
| **SAL_278** | **Guangxi** | **Human** | **MDR** | **2015** | **SAL00278** |
| **SAL_279** | **Guangxi** | **Human** | **MDR** | **2015** | **SAL00276** |
| **SAL_280** | **Guangxi** | **Human** | **SDR** | **2015** | **SAL00280** |
| **SAL_281** | **Guangxi** | **Human** | **MDR** | **2015** | **SAL00281** |
| **SAL_282** | **Guangxi** | **Human** | **MDR** | **2015** | **SAL00282** |
| **SAL_283** | **Guangxi** | **Human** | **MDR** | **2015** | **SAL00283** |
| **SAL_284** | **Guangxi** | **Human** | **MDR** | **2015** | **SAL00284** |
| **SAL_285** | **Guangxi** | **Human** | **MDR** | **2015** | **SAL00285** |
| **SAL_286** | **Guangxi** | **Human** | **SDR** | **2015** | **SAL00286** |
| **SAL_287** | **Shanghai** | **Human** | **MDR** | **2016** | **SAL00287** |
| **SAL_288** | **Shanghai** | **Human** | **SDR** | **2016** | **SAL00288** |
| **SAL_289** | **Shanghai** | **Human** | **MDR** | **2016** | **SAL00289** |
| **SAL_290** | **Fujian** | **Human** | **MDR** | **2016** | **SAL00290** |
| **SAL_291** | **Shanghai** | **Human** | **MDR** | **2016** | **SAL00291** |
| **SAL_292** | **Shanghai** | **Human** | **MDR** | **2016** | **SAL00292** |
| **SAL_293** | **Shanghai** | **Human** | **SDR** | **2016** | **SAL00293** |
| **SAL_294** | **Shanghai** | **Human** | **SDR** | **2016** | **SAL00294** |
| **SAL_295** | **Shanghai** | **Human** | **MDR** | **2016** | **SAL00295** |
| **SAL_296** | **Shanghai** | **Human** | **SDR** | **2016** | **SAL00296** |
| **SAL_297** | **Shanghai** | **Human** | **SDR** | **2016** | **SAL00297** |
| **SAL_298** | **Shanghai** | **Human** | **SDR** | **2016** | **SAL00298** |
| **SAL_299** | **Shanghai** | **Human** | **SDR** | **2016** | **SAL00299** |
| **SAL_300** | **Shanghai** | **Human** | **MDR** | **2016** | **SAL00300** |
| **SAL_301** | **Shanghai** | **Human** | **SDR** | **2016** | **SAL00301** |
| **SAL_302** | **Zhejiang** | **Human** | **MDR** | **2016** | **SAL00302** |
| **SAL_303** | **Fujian** | **Human** | **MDR** | **2016** | **SAL00303** |
| **SAL_304** | **Shanghai** | **Human** | **MDR** | **2016** | **SAL00304** |
| **SAL_305** | **Chongqing** | **Human** | **MDR** | **2016** | **SAL00305** |
| **SAL_306** | **Chongqing** | **Human** | **MDR** | **2016** | **SAL00306** |
| **SAL_307** | **Chongqing** | **Human** | **SDR** | **2016** | **SAL00307** |
| **SAL_308** | **Guangxi** | **Human** | **MDR** | **2007** | **SAL00308** |
| **SAL_309** | **Guangxi** | **Human** | **MDR** | **2009** | **SAL00309** |
| **SAL_310** | **Guangxi** | **Human** | **SDR** | **2013** | **SAL00310** |
| **SAL_311** | **Guangxi** | **Human** | **MDR** | **2014** | **SAL00311** |
| **SAL_312** | **Guangxi** | **Human** | **MDR** | **2014** | **SAL00312** |
| **SAL_313** | **Guangxi** | **Human** | **MDR** | **2016** | **SAL00313** |
| **SAL_314** | **Guangxi** | **Human** | **MDR** | **2016** | **SAL00314** |
| **SAL_315** | **Guangxi** | **Human** | **SDR** | **2016** | **SAL00315** |
| **SAL_316** | **Guangxi** | **Human** | **SDR** | **2016** | **SAL00316** |
| **SAL_317** | **Guangxi** | **Human** | **SDR** | **2016** | **SAL00317** |
| **SAL_318** | **Hubei** | **Human** | **SDR** | **2013** | **SAL00318** |
| **SAL_319** | **Hubei** | **Human** | **MDR** | **2013** | **SAL00319** |
| **SAL_320** | **Hubei** | **Human** | **MDR** | **2012** | **SAL00320** |
| **SAL_321** | **Hubei** | **Human** | **MDR** | **2012** | **SAL00321** |
| **SAL_322** | **Hubei** | **Human** | **SDR** | **2013** | **SAL00322** |
| **SAL_323** | **Hubei** | **Human** | **Pan-susceptible** | **2014** | **SAL00323** |
| **SAL_324** | **Hubei** | **Human** | **SDR** | **2014** | **SAL00324** |
| **SAL_325** | **Hubei** | **Human** | **MDR** | **2014** | **SAL00325** |
| **SAL_326** | **Hubei** | **Human** | **SDR** | **1997** | **SAL00326** |
| **SAL_327** | **Shanghai** | **Human** | **MDR** | **2016** | **SAL00327** |
| **SAL_328** | **Hubei** | **Human** | **SDR** | **2016** | **SAL00328** |
| **SAL_329** | **Hubei** | **Human** | **MDR** | **2016** | **SAL00329** |
| **SAL_330** | **Hubei** | **Human** | **SDR** | **2016** | **SAL00330** |
| **SAL_331** | **Hubei** | **Human** | **SDR** | **2016** | **SAL00331** |
| **SAL_332** | **Shanxi** | **Human** | **MDR** | **2013** | **SAL00332** |
| **SAL_333** | **Guangxi** | **Human** | **MDR** | **2013** | **SAL00333** |
| **SAL_334** | **Guangxi** | **Human** | **SDR** | **2013** | **SAL00334** |
| **SAL_335** | **Guangxi** | **Human** | **SDR** | **2013** | **SAL00335** |
| **SAL_336** | **Guangxi** | **Human** | **SDR** | **2014** | **SAL00336** |
| **SAL_337** | **Guangxi** | **Human** | **MDR** | **2014** | **SAL00337** |
| **SAL_338** | **Guangxi** | **Human** | **MDR** | **2014** | **SAL00338** |
| **SAL_339** | **Guangxi** | **Human** | **SDR** | **2014** | **SAL00339** |
| **SAL_340** | **Guangxi** | **Human** | **MDR** | **2014** | **SAL00340** |
| **SAL_341** | **Shenzhen** | **Human** | **SDR** | **2007** | **SAL00341** |
| **SAL_342** | **Shenzhen** | **Human** | **MDR** | **2008** | **SAL00342** |
| **SAL_343** | **Shenzhen** | **Human** | **Pan-susceptible** | **2009** | **SAL00343** |
| **SAL_344** | **Shenzhen** | **Human** | **SDR** | **2010** | **SAL00344** |
| **SAL_345** | **Shenzhen** | **Human** | **Pan-susceptible** | **2010** | **SAL00345** |
| **SAL_346** | **Shenzhen** | **Human** | **MDR** | **2011** | **SAL00346** |
| **SAL_347** | **Shenzhen** | **Human** | **MDR** | **2013** | **SAL00347** |
| **SAL_348** | **Shenzhen** | **Human** | **MDR** | **2013** | **SAL00348** |
| **SAL_349** | **Shenzhen** | **Human** | **MDR** | **2014** | **SAL00349** |
| **SAL_350** | **Shenzhen** | **Human** | **SDR** | **2015** | **SAL00350** |
| **SAL_351** | **Shenzhen** | **Human** | **SDR** | **2017** | **SAL00351** |
| **SAL_352** | **Shenzhen** | **Human** | **MDR** | **2017** | **SAL00352** |
| **SAL_353** | **Zhejiang** | **Human** | **MDR** | **2018** | **SAL01684** |
| **SAL_354** | **Zhejiang** | **Human** | **MDR** | **2018** | **SAL01720** |
| **SAL_355** | **Zhejiang** | **Human** | **MDR** | **2018** | **SAL01722** |
| **SAL_356** | **Zhejiang** | **Human** | **MDR** | **2018** | **SAL01738** |

N.B. Yellow color refers to colistin resistant isolates in this study. The antimicrobials used includes ampicillin; gentamicin; kanamycin, tetracycline; ciprofloxacin; chloramphenicol; sulfamethoxazole-trimethoprim; amoxicillin-clavulanic acid; ceftriaxone; cefoxitin; nalidixic acid; colistin; meropenem. Strains were categorized into three antibiotic categories: pan‐susceptible, simple‐drug‐resistance (SDR) defined as one or two types of drug resistance, or multi‐drug‐resistance (MDR) defined as resistance to drugs of at least three different antimicrobial classes.

**Supplemental Table 2. Metadata for the Available 24 Chinese Isolates Included in Phylogenomic Analysis.**

| **Isolate ID** | **Accession number** | **Source** | **Place of Origin** | **Year of isolation** |
| --- | --- | --- | --- | --- |
| ATCC-27869  CFSAN014184*  CFSAN014956*  CFSAN068091*  CFSAN068092*  FNE0040*  Henan_3  Shandong_3  JS09102  SH111077  SHSN001  SHSN002  SHSN003  SHSN004  SHSN005  SHSN006  SHSN007  SHSN008  SHSN009  SHSN010  SHSN011  SHSN012  SHSN013  SHSN014 | SAMN07500518  SAMN02844004  SAMN02844776  SAMN07560051  SAMN07560050  SAMN02345181  SAMN02415263  SAMN02415264  SAMN02415261  SAMN02415262  SAMN06677850  SAMN06677849  SAMN06677848  SAMN06677847  SAMN06677862  SAMN06677861  SAMN06677859  SAMN06677860  SAMN06677858  SAMN06677857  SAMN06677856  SAMN06677855  SAMN06677854  SAMN06677846 | Laboratory  Tilapia  Frog  Frozen Octopus Frozen Octopus  Orange  Human  Human  Human  Human  Food  Human  Turtle  Chicken meat  Scallop  Pig  Water  Duck  Duck  Scallop  Duck  Human  Human  Human | USA  China  China  China  China  China  China:Henan  China:Shandong  China:Jiangsu  China:Shanghai  China:Shanghai  China:Guangdong  China:Shanghai  China:Shanghai  China:Shanghai  China:Shanghai  China:Shanghai  China:Shanghai  China:Shanghai  China:Shanghai  China:Shanghai  China:Shanghai  China:Shanghai  China:Shanghai | 2004  2009  2017  2017  2011  2013  2013  2010  2011  2012  2012  2011  2011  2011  2008  2010  2010  2009  2010  2008  2011  2010  2009 |

* These isolates collected by U.S. FDA under importing food inspection and only mentioned China as their origin without mentioning the specific province.

| **Isolate**  **(ID)** | **year** | **Origin** | **Source** | **Health**  **status** | **Colistin (MIC**  **mg/L)** | **MLST** | **Phenotypic resistance** | **Resistance genes** | **Chromosomal point mutations** | **PEA**  **Gene** |
| --- | --- | --- | --- | --- | --- | --- | --- | --- | --- | --- |
| ***Sal_***  **276** | 2016 | Guangxi | Feces | Occupational  examination | 16 | 31 | CST, AMC, CIP, GEN, KAN, COT, TET, NAL and CHL. | *tet*(M), *aph(4)-Ia*, *aph*(3')*-Ia*, *floR*, *aac(6')-Iaa*, *aac(3)-Iva*, *qnrS1*,*tet*(A) | ND | *mcr*-1 |
| ***Sal_***  **311** | 2015 | Guangxi | Feces | Diarrhea  (patient) | 16 | 31 | CST, AMC, GEN, AMP, TIO, CRO, Fox, COT, TET, NAL and CHL. | *tet*(M), *aph(4)-Ia*, *aph*(3')*-Ia*, *floR*, *bla*_TEM-1C_, *aac(6')-Iaa*, *aac(3)-Iva* | ND | *mcr*-1 |
| ***Sal_***  **353** | 2017 | Zhejiang | Feces | Diarrhea  (patient) | 4 | 46 | CST, GEN | *aac(6')-Iaa* | *pmrB* (A181S) | *EptC* |
| ***Sal_***  **354** | 2017 | Zhejiang | Feces | Diarrhea  (patient) | 4 | 45 | CST, GEN, CIP, COT, TET, FOX, AMP, NAL, TIO, AMC, KAN | *tet*(B), *bla*_OX-1_, *aac (6')-Iaa* , *aac (6)-Ib-cr*, *qnrA1*, *catB3*, *arr-3*, *sul-1*, *aph(3')-Ia* | *pmrA* (V30F) *pmrA* (A33V) | *EptC* |

Supplemental Table 3. Detail Information about the Chinese colistin-resistant strains identified in this study

AMP, ampicillin; GEN, gentamicin; KAN, kanamycin, TET, tetracycline; CIP, ciprofloxacin; CHL, chloramphenicol; COT, sulfamethoxazole-trimethoprim; AMC, amoxicillin-clavulanic acid; TIO, ceftriaxone; FOX, cefoxitin; NAL, nalidixic acid; CST, colistin.
